# The Prevalence and Trend of Metabolic Syndrome in the South-East of Iran

**DOI:** 10.25122/jml-2020-0052

**Published:** 2020

**Authors:** Khadijeh Kalan Farmanfarma, Mahmoud Ali Kaykhaei, Mehdi Mohammadi, Hussein Ali Adineh, Alireza Ansari-Moghaddam

**Affiliations:** 1. Department of Epidemiology & Biostatistics, Health Promotion Research Center, Zahedan University of Medical Sciences, Zahedan, Iran; 2. Department of Epidemiology and Biostatistics, School of Health, Iranshahr University of Medical Sciences, Iranshahr, Iran

**Keywords:** Prevalence, trend, metabolic syndrome, Iran

## Abstract

Metabolic syndrome (Mets) is a set of metabolic disorders including abdominal obesity, insulin resistance or disorders of glucose absorption and metabolism, lipid disorders, and hypertension, which increases the risk of chronic diseases including type 2 diabetes, cardiovascular diseases, cancer, and mortality. Therefore, the present study aimed to determine the trend of Mets and its components in Zahedan, a city located in South-East of Iran, from 2009 to 2017. A total of 761 individuals aged >20 years were followed from 2009 to 2017. The frequency of metabolic syndrome was measured at two-time points based on four criteria: International Diabetes Federation (IDF), National Cholesterol Education Program-Third Adult Treatment Panel (NCEP-ATP III), Third Adult Treatment Panel (ATP III), and American Heart Association and the National Heart Lung and Blood Institute (AHA/NHLBI). The prevalence of Mets ranged from 16.6 (95% CI: 13.9 - 19.2) (ATP III) to 23.70% (95% CI: 20.6 - 26.6) (AHA/NHLBI) in 2009. Accordingly, it increased from 5.2% to 7.3% during the study period using different criteria such that the frequency of Mets varied from 21.8 (95% CI: 18.8 - 24.7) (ATP III) to 31.0% (95% CI: 27.7 - 34.3) (AHA/NHLBI) in 2017. The increasing trend was prominent among females, persons aged <40 years, and those with the lowest educational level. Two components of Mets (abdominal obesity and diabetes) increased in prevalence, whereas elevated blood pressure, hypertriglyceridemia, and low HDL declined. The study revealed an annual increase rate of about 1% in the prevalence of metabolic syndrome. Therefore, the increasing trend of some components of Mets highlights the urgency of addressing these components as health priorities.

## Introduction

Metabolic syndrome (Mets) is a set of metabolic disorders including abdominal obesity, insulin resistance or disorders of glucose absorption and metabolism, lipid disorders, and hypertension [1], which increases the risk of chronic diseases including type 2 diabetes, cardiovascular diseases, cancer, and mortality [2]. Therefore, along with the epidemics of cardiovascular risk factors, Mets becomes a major challenge for global public health [3]. According to recent estimates, on average, 20 to 30% of the adult population in most countries [4] suffer from Mets. Nevertheless, the prevalence rate of Mets has been reported variously across the world. For example, data suggest a frequency of 24.3% in 10 European countries [5], 27.21% in Turkey [6], and 30.0% in Bangladesh [7]. Accordingly, based on various diagnostic criteria, a range of 13 -37% of the Iranian population are now living with Mets [8].

There is some evidence that behavioral and environmental changes, such as the adoption of Western diets and lifestyle, lack of physical activity, and improved socioeconomic status in developing countries, might be the possible major causes of the Mets pandemic [1]. In addition to these factors, the aging of the population, increased obesity prevalence, and glucose intolerance can also accelerate the growing trend of Mets in both developed and developing regions [8]. Consequently, the world could witness an escalating trend of Mets in the near future if the current trend continues without any changes. For instance, a study demonstrated that the frequency of Mets has increased from 32.9% to 34.7% from 2003 to 2012 in the US [9]. Importantly, to the best of our knowledge, no study has examined the trend of Mets in the South-Eastern region of Iran. Therefore, the present study aimed to determine the trend of Mets and its components in Zahedan, a city located in the South-East of Iran from 2009 to 2017.

## Material and Methods

### Study design, setting and population

The present epidemiological cohort study was conducted on the urban population aged >20 years of Zahedan, Iran. A total of 761 people who were enrolled and examined in a study in 2009 have been followed after an eight-year interval and have been re-examined in 2017 [10, 11]. The inclusion criteria were age >20 years, being of Iranian nationality, absence of intellectual disability, hemorrhagic diseases, cardiovascular diseases, and psychiatric disorders, as well as not taking mineral supplements or drugs that affect the metabolism of nutrients and blood lipids in 2009. The same people were reviewed again in 2017. After coordination and approval by the Deputy of Research and Technology of Zahedan University of Medical Sciences, records of the study sample in 2009 were reviewed. Individuals were gradually contacted and invited to participate in the study. After that, their houses were visited, and they have been given explanations regarding the objectives of the study. It was also explained that their participation is voluntary, and all the collected data would remain confidential.

A cross-sectional study was carried on a total of 1802 participants from September 2008 to March 2009 in Zahedan, a city in the South-East of Iran. Details regarding sample size calculation are explained elsewhere [10]. Accordingly, the sample size of the 2017 study were all participants of the 2009 study who were available at the time of follow-up (n = 761).

### Sampling method and participants recruitment

To include a representative sample, a multistage random sampling method was used. Firstly, the city of Zahedan was divided into 20 strata based on the division of the map in the civil registry with the cooperation of the Civil Registration Organization. Then, samples were selected by random cluster sampling from the 20 aforementioned regions of Zahedan.

The same population was followed-up again in 2017. At first, the research team contacted the subjects of the study by referring to their profile in 2009. Then, a trained team, including a laboratory expert and interviewers, visited individuals’ homes based on appointed times. Consequently, researchers collected blood samples and completed questionnaires from individuals in their residential areas. In 2017, we reached only 761 individuals because of population movements despite inspecting all available profiles (n= 1802) and following them. Nevertheless, the missing number was approximately similar in all clusters, and the surveyed people were a nearly good representative of the city.

### Data collection and study procedure

Three questionnaires were used to collect data including the International Physical Activity Questionnaires (IPAQ), a researcher-made dietary questionnaire with a content validity ratio (CVR) = 0.77, a content validity index (CVI) = 0.87 and reliability of 0.71 as well as researcher-made a questionnaire for smoking and tobacco with CVR = 0.75, CVI = 0.83 and reliability of 0.71.

After obtaining written consent forms and upon their approval, the aforementioned questionnaires were completed by trained individuals during face-to-face interviews. Moreover, anthropometric indices (including height, weight, waist circumference, and blood pressure) were measured based on a standard plan. Weight was measured while the participants were wearing light clothing and no shoes on scales (Seca) with a precision of 100 g. Height was measured using a stadiometer (Seca), standing and without shoes, with shoulders in normal positions, with a precision of 1 cm. To determine abdominal obesity, waist circumference was measured in its narrowest point, when the person was at the end of his/her natural exhalation, using a stretch-resistant cloth tape without any pressure on the body and with a precision of 0.1 cm. Moreover, blood pressure was measured twice using a standard sphygmomanometer with an appropriate arm cuff placed on the right arm after the person had been seated for 15 minutes. The average of two measurements was calculated and represented the final blood pressure.

### Blood sampling and blood assay

Then, participants were asked to be ready for blood sampling collection the following day. A fasting blood sample was collected from each of the participants in the morning of the day after the interview. The blood samples of all participants were centrifuged to separate the serum. It was initially stored at -20°C and then at -80°C. Then, serum glucose, triglyceride, and cholesterol levels were measured using calorimetric methods via the Biotech kit and ELAN 2000 auto-analyzer device, and HDL-C and LDL levels were measured via direct methods.

### Definition of metabolic syndrome

Four criteria were used to identify metabolic syndrome, as shown in Table 1.

**Table 1:** Criteria for diagnosis of metabolic syndrome [12].

**Criterion**	† NCEP-ATP III	*IDF	† AHA/NHLBI	† ATP III
**Waist Circumference (cm)**				
Male Female	≥102 ≥88	≥94 ≥80	≥94 ≥80	≥102 ≥88
**HDL (mg/dl)**				
Male Female	<40 <50	<40 <50	<40 <50	<40 <50
**Triglyceride (mg/dl)**	≥150	≥150	≥150	≥150
**Fasting Glucose (mg/dl)**	≥100	≥100	≥100	≥110
**Blood Pressure (mmHg)**	≥130/85	≥130/85	≥130/85	≥130/85

* - Central adiposity required; two of the subsequent four are required; † - Three of five are required. AHA-NHLBI: American Heart Association and the National Heart Lung and Blood Institute; ATP III: Third Adult Treatment Panel; IDF: International Diabetes Federation; NCEP-ATP III: National Cholesterol Education Program-Third Adult Treatment Panel.

### Statistical analysis

Data were described and analyzed using the SPSS software, version 16. Frequency distribution, mean, and standard deviation were used to summarize data. Association between variables was examined using the Chi-square test, independent t-test, and multiple logistic regression analysis at a significance level of 0.05.

## Results

In the present study, 761 people (50.9% women and 49.1% men) were examined in two periods. The mean age of participants was 38.39 ± 12.54 years at the beginning of the study and 46.86 ± 12.47 years at the end of the follow-up, which was significantly different (P < 0.001). 56.6% of the target population were Sistani, Baluch (30.5%), Birjandi (8.7%), and 4.2% had other ethnicities. Regarding the education level, approximately half of them were illiterate (18.3%) or below high school diploma (32.7%). 18.3% of them had a high school diploma, and the remaining subjects had an academic degree (30.7%). In the second study period, that percentage significantly promoted towards a high level compared to the first study point (P < 0.001). Moreover, 88% of individuals had high-risk occupations, compared to 12% that had a low-risk occupation at the baseline. High-risk jobs included jobs where the person has less physical activity and has to sit for a long time, like office jobs; low-risk jobs included jobs that have more physical activity, such as agriculture. Also, the percentages of subjects with high-risk changed meaningfully to 97.9% in the second study period.

### Prevalence of metabolic syndrome

The frequency of Mets was estimated based on four criteria, including International Diabetes Federation (IDF), National Cholesterol Education Program-Third Adult Treatment Panel (NCEP-ATP III), Third Adult Treatment Panel (ATP III), and American Heart Association and the National Heart Lung and Blood Institute (AHA/NHLBI). Prevalence of Mets ranged from 16.6 (95% CI: 13.92 - 19.2) (ATP III) to 23.70% (95% CI: 20.63 - 26.67) (AHA/NHLBI) according to the different definitions from 2009 (Table 2). In comparison, it increased during a time interval of 8 years between 5.2% to 7.3% based on the four used criteria, such that the frequency of Mets varied from 21.8 (95% CI: 18.88 - 24.75) (ATP III) to 31.0% (95% CI: 27.73 - 34.3) (AHA/NHLBI) in 2017. Moreover, based on NCEP-ATP III, it increased from 17.9% (95% CI: 15.15 - 20.59) in 2009 to 24.3% (95% CI: 21.26 - 27.36) in 2017 (by 6.4%).

**Table 2:** Prevalence of metabolic syndrome by sex, age, year of study and diagnostic criteria.

Criteria Variable		IDF		NCEP-ATP III		ATP III		AHA-NHLBI	
		2009 Prevalence (95% CI)	2017 Prevalence (95% CI)	2009 Prevalence (95% CI)	2017 Prevalence (95% CI)	2009 Prevalence (95% CI)	2017 Prevalence (95% CI)	2009 Prevalence (95% CI)	2017 Prevalence (95% CI)
**Sex**	**Male**	16.6 (12.8 – 20.3)	19.8 (15.7 -23.8)	11.3 (8.0 -14.4)	13.4 (9.9-16.8)	10.2 (7.1 -13.2)	11.2 (8.0 -14.4)	18.5 (14.5 – 22.3)	22.2 (17.9 – 26.4)
	**Female**	28.8 (24.1 – 33.1)	35.9 (31.1 – 40.7)	24.4 (20.0 -28.5)	31.5 (26.9 – 36.1)	22.9 (18.5 - 26.9)	28.9 (24.4 -33.4)	28.8 (24.1– 33.1)	36.4 (31.6 – 41.2)
**Age**	<40	14.8 (11.2 – 18.1)	18.1 (13.4 – 22.8)	10.3 (7.2 -13.2)	16.2 (11.7-20.7)	9.3 (6.3 – 12.1)	13.9 (9.6 – 18.1)	15.9 (12.2 – 19.5)	20.8 (15.9 -25.8)
	**≤** **≥40**	31.7 (26.7 – 36.3)	34.7 (30.5 – 38.9)	26.4 (21.7 -30.8)	28.5 (24.5 – 32. 5)	24.7 (20.1 – 29.0)	25.9 (22.1- 29.7)	32.6 (27.5– 37.2)	36.3 (32.1 - 40.5)
**Total**		22.8 (19.7 – 25.7)	9.0 (25.8 – 32.2)	17.9 (15.1 -20.5)	24.3 (21.2– 27.3)	16.6 (13.9 - 19.2)	21.8 (18.8 – 24.7)	23.7 (20.6 – 26.6)	31.0 (27.7 -34.3)

AHA-NHLBI: American Heart Association and the National Heart Lung and Blood Institute; ATP III: Third Adult Treatment Panel; IDF: International Diabetes Federation; NCEP-ATP III: National Cholesterol Education Program—Third Adult Treatment Panel.

Importantly, the frequency of Mets was significantly higher among women compared to men in both periods of time based on all four criteria (Table 2). It varied from 22.9% (95% CI: 18.56 - 26.92) (ATP III) to 28.8% (95% CI: 24.18 - 33.19) (AHA/NHLBI) amongst females in 2009. Accordingly, it increased by 6% to 7.6% based on a different definition during a period of 8 years such that the prevalence of Mets ranged from 28.9% (95% CI: 24.42 - 33.46) (ATP III) to 36.4% (95% CI: 31.64 - 41.23) (AHA/NHLBI) 2017. In comparison, a fraction of 10.2% (95% CI: 7.1 - 13.22) (ATP III) to 18.5% (95% CI: 14.52 - 22.38) (AHA/NHLBI) of males were suffering from Mets in 2009, which increased by 1% to 3.7% during the two periods. Therefore, a proportion of 11.2% (95% CI: 8.03 - 14.43) (ATP III) to 22.2% (95% CI: 17.98 - 26.4) (AHA/NHLBI) of males were classified as suffering from Mets in 2017.

The prevalence of Mets was approximately two times higher in individuals aged ≥40 years than those aged <40 years based on different diagnostic criteria and two study periods (Table 2). Indeed, the frequency of Mets changed from 24.7% (95% CI: 20.12 - 29.04) (ATP III) to 32.6% (95% CI: 27.55 - 37.25) (AHA/NHLBI) among subjects aged ≥40 years in 2009, showing an increase of 1.2% to 3.7% during the two study periods. Comparably, a range of 9.30% (95% CI: 6.36 - 12.1) to 15.9% (95% CI: 12.27 - 19.53) individuals less than 40 years old had Mets in 2009 according to various definitions. Interestingly, Mets prevalence increased by 4.6% to 5.9% among cases aged <40 years based on different criteria.

**Figure 1:**
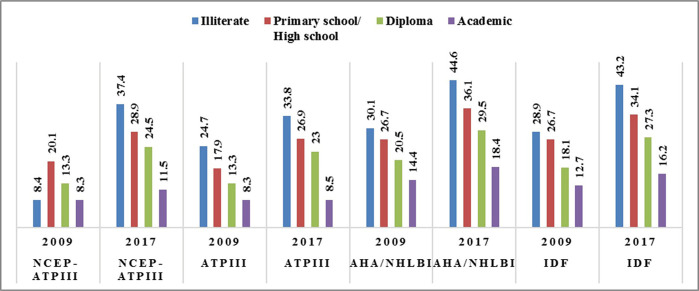
Prevalence of metabolic syndrome in the total population according to education. AHA-NHLBI: American Heart Association and the National Heart Lung and Blood Institute; ATP III: Third Adult Treatment Panel; IDF: International Diabetes Federation; NCEP-ATP III: National Cholesterol Education Program—Third Adult Treatment Panel.

There has been an inverse association between changes in the frequency of Mets during the study period and the educational level of participants (Figure 1). The changes in the frequency of Mets from 2009 to 2017 ranged from 0.2% (ATP III) to 4% (AHA/NHLBI) in those with an academic degree. In comparison, those with the lowest educational level/ illiterate experienced an increase of 9.1% (ATP III) to 29% (NCEP-ATP III) in the prevalence of Mets, which is 3 to more than 10 times higher than that of the highest education level.

### Components of metabolic syndrome

Table 3 shows the prevalence trend in the overall components of Mets and by gender. Generally, two out of five components of Mets (abdominal obesity and diabetes) increased in prevalence, and three of them, including elevated blood pressure, hypertriglyceridemia, and low HDL levels, declined. Nevertheless, the trends of Mets components varied between males and females during the study period.

**Table 3:** Prevalence of the components of metabolic syndrome by sex, year and diagnostic criteria.

Criteria Variable		IDF		NCEP-ATP III		ATP III		AHA-NHLBI	
		**2009 Prevalence (95% CI)**	2017 Prevalence (95% CI)	2009 Prevalence (95% CI)	2017 Prevalence (95% CI)	2009 Prevalence (95% CI)	2017 Prevalence (95% CI)	2009 Prevalence (95% CI)	2017 Prevalence (95% CI)
Waist Circumference	Male	42.5 (37.4 - 47.5)	44.0 (38.9 – 49.0)	18.8 (14.8 – 22.7)	16.4 (12.6 – 20.1)	18.8(14.8 – 22.7)	16.4 (12.6 – 20.1)	42.5 (37.4 - 47.5)	44.0 (38.9 – 49.0)
	**Female**	68.8 (64.1 – 73.4)	79.3 (75.2 – 83.3)	47.1 (42.1 – 50.0)	62.2 (57.3 – 67.0)	47.1 (42.1 – 50.0)	62.2 (57.3 – 67.0)	68.8 (64.1 – 73.4)	79.3 (75.2 – 83.3)
	**Total**	55.5 (52.3 -59.4)	61.9 (58.5 - 65.4)	33.1 (29.8 -36.5)	39.6 (36.2 - 43.1)	33.1 (29.8 -36.5)	39.6 (36.2 - 43.1)	55.5 (52.3 -59.4)	61.9 (58.5 - 65.4)
Blood Pressure	Male	35.8 (30.9 – 40.6)	29.4 (24.7 – 34.0)	35.8 (30.9 – 40.6)	29.4 (24.7 – 34.0)	35.8 (30.9 – 40.6)	29.4 (24.7 – 34.0)	35.8 (30.9 – 40.6)	29.4 (24.7 – 34.0)
	**Female**	22.7 (18.5 – 26.8)	26.1 (21.7 – 30.4)	22.7 (18.5 – 26.8)	26.1 (21.7 – 30.4)	22.7 (18.5 – 26.8)	26.1 (21.7 – 30.4)	22.7 (18.5– 26.8)	26.1 (21.7 – 30.4)
	**Total**	29.1 (25.8 – 32.3)	27.7 (24.5 – 30.8)	29. 1 (25.8 – 32.3)	27.7 (24.5 – 30.8)	29.1 (25.8 – 32.3)	27.7 (24.5 – 30.8)	29.1 (25.8 – 32.3)	27.7 (24.5 – 30.8)
Triglycerides	Male	44.6 (39.5 – 49.6)	36.1 (31.2 – 40.9)	44.6 (39.5 – 49.6)	36.1 (31.2 – 40.9)	44.6 (39.5 – 49.6)	36.1 (31.2 – 40.9)	44.6 (39.5 – 49.6)	36.1 (31.2 – 40.9)
	**Female**	29.9 (25.3 – 34.4)	30.5 (25.9 – 35.0)	29.9 (25.3 – 34.4)	30.5 (25.9 – 35.0)	29.9 (25.3 – 34.4)	30.5 (25.9 – 35.0)	29.9 (25.3 – 34.4)	30.5 (25.9 – 35.0)
	**Total**	37.1 (33.6 – 40.5)	33.2 (29.8 – 36.5)	37.1 (33.6 – 40.5)	33.2 (29.8 – 36.5)	37.1 (33.6 – 40.5)	33.2 (29.8 – 36.5)	37.1 (33.6 – 40.5)	33.2 (29.8 – 36.5)
HDL-C	Male	14.4 (10.8 – 17.9)	16.8 (13.0 - 20.5)	14.4 (10.8 – 17.9)	16.8 (13.0 - 20.5)	14.4 )10.8 – 17.9)	16.8 (13.0 - 20.5)	14.4 (10.8 – 17.9)	16.8 (13.0 - 20.5)
	**Female**	66.9 (62.2 – 71.5)	46.1 (41.1 – 51.0)	66.9 (62.2 – 71.5)	46.1 (41.1 – 51.0)	66.9(62.2 – 71.5)	46.1 (41.1 – 51.0)	66.9 (62.2 – 71.5)	46.1 (41.1 – 51.0)
	**Total**	41.09 (37.5 -44.5)	31.7 (28.3 – 35.0)	41.0 (37.5 - 44.5)	31.7 (28.3 – 35.0)	41.0 (37.5 - 44.5)	31.7 (28.3 – 35.0)	41.0 (37.5 - 44.5)	31.7 (28.3– 35.0)
Glucose	Male	15.5 (11.8 – 19.1)	26.2 (21.7 – 30.6)	15.5 (11.8 – 19.1)	26.2 (21.7 – 30.6)	10.6 (7.4 – 13.7)	16.6 (12.8 – 20.3)	15.5 (11.8 – 19.1)	26.2 (21.7 – 30.6)
	**Female**	12.1 (8.8 – 15.3)	26.1 (21.7 – 30.4)	12.1 (8.8– 15.3)	26.1 (21.7 – 30.4)	7.60 (4.9 – 10.2)	18.1(14.2 – 21.9)	12.1 (8.8 – 15.3)	26.1 (21.7 – 30.4)
	**Total**	13.8 (11.35 – 16.25)	26.1 (22.98-29.22)	13.8 (11.35 – 16.25)	26.1 (22.9-29.22)	9.10 (7.0 – 11.1)	17.3 (14.6 -19.9)	13.8 (11.3 – 16.2)	26.1(22.9 -29.2)

AHA-NHLBI: American Heart Association and the National Heart Lung and Blood Institute; ATP III: Third Adult Treatment Panel; IDF: International Diabetes Federation; NCEP-ATP III: National Cholesterol Education Program—Third Adult Treatment Panel.

• Waist circumference: Abdominal obesity increased by 6.8% to 6.5% (net increase) from 2009 to 2017 in the total population based on the different criteria which were prominent in females (a net increase of 10.5% to 15.2% in prevalence rates compared to 1.5% to 2.4% in males according to the different definitions). Importantly, the baseline rates of waist circumference were much higher in females than males.
• Blood glucose: The proportion of individuals with blood glucose increased significantly in both genders, with a greater increase in women. It has risen from 9.1 (95% CI: 7.06 - 11.14) in 2009 to 17.3% (95% CI: 14.61 - 19.99) in 2017 based on ATP III and from 13.8 (11.35 - 16.25) to 26.1 (22.98 - 29.22) in accordance with the other criteria. The net increase varied from 10.5% (ATP III) to 14% (other definitions) among females compared to a range of 6% (ATP III) to 10.7% (other definitions) in males.
• Blood pressure: In general, the frequency of blood pressure declined slightly from 29.1% (95% CI: 25.87 - 32.33) to 27.7% (95% CI: 24.52 – 30.88) over time. Nevertheless, the trend was contrasted in terms of gender such that women experienced a 3.4% increase of elevated blood pressure, whereas a decreasing trend of 5.4% was seen amongst men.
• Triglycerides: The prevalence of individuals meeting the hypertriglyceridemia criterion decreased during the study period from 37.1% (95% CI: 33.67 to 40.53) to 33.2% (95% CI: 29.85 - 36.55). Again, a diverse trend was seen in the two sexes. Hypertriglyceridemia decreased by 7.5% in males compared to an approximately 1% increase amongst females.
• HDL-Cholesterol: A reduction trend was observed in the prevalence of low HDL-cholesterol over time in the total population from 41.09% (95% CI: 37.59 - 44.59) to 31.70% (95% CI: 28.39 - 35.01) and prominently in women [from 66.9% (95% CI: 62.21 - 71.59) to 46.1% (95% CI: 41.13 - 51.07)]. In contrast, the low HDL level increased over time by 2.4% in men.

### Comparison of the components in the two study periods

Table 4 shows variations in Mets components between the two study periods in which changes in Mets components were significant (P < 0.01) except for blood pressure and HDL cholesterol. After the study period, the most significant changes toward abnormal were observed in blood sugar and waist circumference compared to the lowest ones in the HDL level (2.51%).

**Table 4:** Comparison of the components of metabolic syndrome before and after follow-up.

** **	N	Without change from 2009 to 2017		Change from normal in 2009 to abnormal in 2017		Change from abnormal in 2009 to normal in 2017		P-Value Mc-Nemar
** **		N	%	N	%	N	%	
**Blood Pressure**	755	503	66.62	121	16.03	131	17.35	0.57
**Glucose (100)**	695	510	73.38	136	19.57	49	7.05	<0.001
**Glucose (110)**	695	569	81.87	93	13.38	33	4.75	<0.001
**Triglyceride**	695	524	75.39	69	9.93	102	14.68	0.01
**HDL-C**	676	640	94.67	17	2.51	19	2.81	0.86
**Waist Circumference (94 and 80cm)**	761	540	70.96	135	17.74	86	11.30	0.001
**Waist Circumference (102 and 88 cm)**	761	575	75.56	118	15.51	68	8.93	<0.001

### Predictors of Mets

According to all criteria, women were almost 2 to 3 times more likely to develop Mets than men. Furthermore, the age of individuals significantly varied in patients with Mets than those who did not have Mets based on all criteria. People with illiteracy were significantly more likely (3.57 to 5.46 times) to develop Mets than those with a university degree. In fact, with increasing education, the chances of developing Mets decreased.

In addition, people with high-risk occupations were roughly twice as likely as those with low-risk occupations to develop Mets. Mets components also significantly increased the chances of developing Mets by all criteria. Consequently, blood sugar, triglycerides (TG), HDL cholesterol, waist circumference, and blood pressure levels increased the odds of getting Mets more than 5 times (Tables 5, 6).

**Table 5:** Frequency distribution of demographic variables and components of metabolic syndrome according to the AHA/NHLBI and IDF criteria using logistic regression.

Variable		AHA/NHLBI 2017		OR (95%CI) Univar ate	AHA/NHLBI 2009		OR (95%CI) Univar ate	IDF 2017		OR (95%CI) Univar ate	IDF 2009		OR (95%CI) Univar ate
		Mets N (%)	NO- Mets N (%)		Mets N (%)	NO- Mets N (%)		Mets N (%)	NO- Mets N (%)		Mets N (%)	NO- Mets N (%)	
*Education	Illiterate	62 (44.6)	77 (55.4)	3.57 (2.23 – 5.72)	50 (30.1)	116 (69.9)	2.57 (1.51 -4.37)	60 (43.2)	79 (56.8)	3.91 (2.41 – 6.35)	48 (28.9)	118 (71.1)	2.79 (1.61 – 4.85)
	**Below high school**	90 (36.1)	159 (63.9)	2.51 (1.65 – 3.82)	85 (26.7)	233 (73.3)	2.17 (1.34- 3.52)	85 (34.1)	164 (65.9)	2.67 (1.73 – 4.13)	85 (26.7)	233 (73.3)	2.50 (1.51 – 4.14)
	**Diploma**	41 (29.5)	98 (70.5)	1.85 (1.13 – 3.04)	17 (20.5)	66 (79.5)	1.53 (0.78 – 3.01)	38 (27.3)	101 (72.7)	1.94 (1.16 – 3.23)	15 (18.1)	68 (81.9)	1.51 (0.74 – 3.08)
	**Academic Degree**	43 (18.4)	191 (81.6)	1.00	26 (14.4)	155 (85.6)	1.00	38 (16.2)	196 (83.8)	1.00	23 (12.7)	158 (87.3)	1.00
Sex	Women	147 (38.0)	240 (62.0)	1.96 (1.43 – 2.68)	111 (28.8)	274 (71.2)	1.78 (1.26-2.51)	145 (37.5)	242 (62.5)	2.34 (1.69 -3.25)	111 (28.8)	274 (71.2)	2.03 (1.43 – 2.88)
	**Men**	89 (23.8)	285 (76.2)	1.00	69 (18.5)	304 (81.5)	1.00	76 (20.3)	298 (79.7)	1.00	62 (16.6)	311 (83.4)	1.00
Ethnicity	Baloch	85 (36.3)	147 (63.4)	1.47 (0.65 - 3.34)	59 (25.5)	172 (74.5)	1.02 (0.43 -2.41)	82 (35.3)	150 (64.7)	1.39 (0.61- 3.16)	57 (24.7)	174 (75.3)	0.98 (0.41- 2.30)
	**Sistani**	122 (28.3)	309 (71.7)	1.00 (0.45 - 2.24)	99 (23.1)	330 (76.9)	0.90 (0.39-2.06)	112 (26.0)	319 (74.0)	0.89 (0.40 -1.99)	94 (21.9)	335 (78.1)	0.84 (0.36 -1.93)
	**Brigand**	20 (30.3)	46 (69.7)	1.11 (0.43 – 2.82)	14 (21.2)	52 (78.8)	0.80 (0.29 -2.18)	18 (27.3)	48 (72.7)	0.95 (0.37 -2.45)	14 (21.2)	52 (78.8)	0.80 (0.29 – 2.18)
	**Others**	9 (28.1)	23 (71.9)	1.00	8 (25.0)	24 (75.0)	1.00	9 (28.1)	23 (71.9)	1.00	8 (25.0)	24 (75.0)	1.00
Occupation	High risk	218 (32.5)	452 (67.5)	1.95 (1.13 – 3.35)	51 (22.1)	180 (77.9)	-	206 (30.7)	464 (69.3)	2.24 (1.26 – 4.00)	48 (20.8)	183 (79.2)	-
	**Low risk**	18 (19.8)	73 (80.2)	1.00	0 (0.0)	5 (100.0)	1.00	15 (16.5)	76 (83.5)	1.00	0 (0.0)	5 (100.0)	1.00
Age		Mean ± SD	Mean ± SD	-	Mean ± SD	Mean ± SD	-	Mean ± SD	Mean ± SD	-	Mean ± SD	Mean ± SD	-
	** **	49.67 ± 11.68	45.60 ± 12.62		42.60 ± 10.57	37.05 ± 12.78		49.49 ± 11.13	45.78 ± 12.83		42.87 ± 0.49	37.03 ± 12.76	
Glucose	Abnormal	124 (62.3)	75 (37.7)	6.64 (4.66 -9.46)	68 (70.8)	28 (29.2)	10.56 (6.49 -17.16)	115 (57.8)	84 (42.2)	5.88 (4.14-8.37)	64 (66.7)	32 (33.3)	8.99 (5.60 – 14.42)
	**Normal**	112 (19.9)	450 (80.1)	1.00	112 (18.7)	487 (81.3)	1.00	106 (18.9)	456 (81.1)	1.00	109 (18.2)	490 (81.8)	1.00
TG	Abnormal	80 (72.7)	30 (27.3)	8.46 (5.36-13.35)	67 (50.0)	67 (50.0)	3.96 (2.66 – 5.89)	71 (64.5)	39 (35.5)	6.08 (3.95-9.35)	63 (47.0)	71 (53.0)	3.63 (2.44 – 5.41)
	**Normal**	156 (24.0)	495 (76.0)	1.00	113 (20.1)	448 (79.9)	1.00	150 (23.0)	501 (77.0)	1.00	110 (19.6)	451 (80.4)	1.00
HDL	Abnormal	19 (100.0)	0 (0.0)	-	13 (61.9)	8 (38.1)	4.93 (2.01 – 12.10)	16 (84.2)	3 (15.8)	13.94 (4.02-48.38)	10 (47.6)	11 (52.4)	2.85 (1.18 – 6.83)
	**Normal**	211 (29.2)	512 (70.8)	1.00	167 (24.8)	507 (75.2)	1.00	200 (27.7)	523 (72.3)	1.00	163 (24.2)	511 (75.8)	1.00
Waist circumference	Abnormal	218 (46.3)	253 (53.7)	13.02 (7.81-21.68)	173 (41.0)	249 (59.0)	32.65 (15.06 – 70.76)	218 (46.3)	253 (53.7)	82.43 (26.05 - 260.80)	173 (41.0)	249 (59.0)	-
	**Normal**	18 (6.2)	272 (93.8)	1.00	7 (2.1)	329 (97.9)	1.00	3 (1.0)	287 (99.0)	1.00	0 (0.0)	336 (100.0)	1.00
BP	Abnormal	127 (60.2)	84 (39.8)	6.11 (4.32-8.64)	111 (50.5)	109 (49.5)	6.99 (4.84 – 10.09)	117 (55.5)	94 (44.5)	5.33 (3.77-7.53)	105 (47.7)	115 (52.3)	6.37 (4.41 – 9.21)
	**Normal**	109 (19.8)	441 (80.2)	1.00	68 (12.7)	467 (87.3)	1.00	104 (18.9)	446 (81.1)	1.00	67 (12.5)	468 (87.5)	1.00

AHA-NHLBI: American Heart Association and the National Heart Lung and Blood Institute; ATP III: Third Adult Treatment Panel; IDF: International Diabetes Federation; NCEP-ATP III: National Cholesterol Education Program–Third Adult Treatment Panel.

*P-value the education trend in 2017 according to AHA/NHLBI (< 0.001). P-value for the education trend in 2009 according to AHA/NHLBI (0.003).

*P-value the education trend in 2017 according to IDF (p<0.001). P-value the education trend in 2009 according to IDF (0.001).

**Table 6:** Frequency distribution of demographic variables and components of metabolic syndrome according to the ATP III and NCEP-ATP III criteria using logistic regression.

Variable		ATP III 2017		OR (95%CI) Univar ate	ATP III2009		OR (95%CI) Univar ate	NCEP-ATP III 2017		OR (95%CI) Univar ate	NCEP-ATP III 2009		OR (95%CI) Univar ate
		Mets N (%)	NO- Mets N (%)		Mets N (%)	NO- Mets N (%)		Mets N (%)	NO- Mets N (%)		Mets N (%)	NO- Mets N (%)	
*Education	Illiterate	47 (33.8)	92 (66.2)	5.46 (3.06 – 9.73)	41 (24.7)	125 (75.3)	3.63 (1.92 – 6.85)	52 (37.4)	87 (62.6)	4.58 (2.70-7.77)	44 (26.5)	122 (73.5)	3.99 (2.12 – 7.50)
	**Below high school**	67 (26.9)	182 (73.1)	3.93 (2.30-6.74)	57 (17.9)	261 (82.1)	2.41 (1.32 – 4.40)	72 (28.9)	177 (71.7)	3.11 (1.91 – 5.06)	64 (20.1)	254 (79.9)	2.78 (1.53 – 5.05)
	**Diploma**	32 (23.0)	107 (77.0)	3.20 (1.74 – 5.86)	11 (13.3)	72 (86.7)	1.69 (0.74 – 3.86)	34 (24.5)	105 (75.5)	2.48 (1.42-4.33)	11 (13.3)	72 (86.7)	1.69 (0.74 -3.86)
	**Academic Degree**	20 (8.5)	214 (91.5)	1.00	15 (8.3)	166 (91.7)	1.00	27 (11.5)	207 (88.5)	1.00	15 (8.3)	166 (91.7)	1.00
Sex	Women	118 (30.5)	269 (69.5)	2.97 (2.05 – 4.32)	88 (22.9)	297 (77.1)	2.61 (1.73 – 3.94)	129 (33.3)	258 (66.7)	2.83 (1.99-4.04)	94 (24.4)	291 (75.6)	2.54 (1.71 – 3.78)
	**Men**	48 (12.8)	326 (87.2)	1.00	38 (10.2)	335 (89.8)	1.00	56 (15.0)	318 (85.0)	1.00	42 (11.3)	331 (88.7)	1.00
Ethnicity	Baloch	60 (25.9)	172 (74.1)	1.51 (0.59-3.85)	48 (20.8)	183 (79.2)	1.83 (0.61 – 5.48)	66 (28.4)	166 (71.6)	1.19 (0.51-2.78)	51 (22.1)	180 (77.9)	1.22 (0.47 – 3.14)
	**Sistani**	85 (19.7)	346 (80.3)	1.06 (0.42-2.66)	64 (14.9)	365 (85.1)	1.22 (0.41 – 3.61)	95 (22.0)	336 (78.0)	0.84 (0.36-1.94)	69 (16.1)	360 (83.9)	0.83 (0.33 – 2.09)
	**Brigand**	15 (22.7)	51 (77.3)	1.27 (0.44-3.67)	10 (15.2)	56 (84.8)	1.25 (0.36 – 4.34)	16 (24.2)	50 (75.8)	0.96 (0.36-2.55)	10 (15.2)	56 (84.8)	0.77 (0.25 – 2.35)
	**Others**	6 (18.8)	26 (81.3)	1.00	4 (12.5)	28 (87.5)	1.00	8 (25.0)	24 (75.0)	1.00	6 (18.8)	26 (81.3)	1.00
Occupation	High risk	155 (23.1)	515 (76.9)	2.18 (1.13-4.21)	30 (13.0)	201 (87.0)	-	173 (25.8)	497 (74.2)	2.29 (1.21-4.30)	33 (14.3)	198 (85.7)	-
	**Low risk**	11 (12.1)	80 (87.9)	1.00	0 (0.0)	5 (100.0)	1.00	12 (13.2)	79 (86.8)	1.00	0 (0.0)	5 (100.0)	1.00
Age		Mean ± SD	Mean ± SD	-	Mean ± SD	Mean ± SD	-	Mean ± SD	Mean ± SD	-	Mean ± SD	Mean ± SD	-
	** **	49.56 ± 11.12	46.11 ± 12.73		43.70 ± 10.92	37.31 ± 12.54		49.63 ± 11.47	45.97 ± 12.66		43.86 ± 10.75	37.17 ± 12.56	
Glucose	Abnormal	76 (57.6)	56 (42.4)	8.12 (5.38)	44 (69.8)	19 (30.2)	15.53 (8.64 -27.90)	105 (52.8)	94 (47.2)	6.73 (4.67-9.69)	61 (63.5)	35 (36.5)	12.17 (7.52 – 19.69)
	**Normal**	90 (14.3)	539 (85.7)	1.00	82 (13.0)	550 (87.0)	1.00	80 (14.2)	482 (85.8)	1.00	75 (12.5)	524 (87.5)	1.00
TG	Abnormal	59 (53.6)	51 (46.4)	5.88 (3.83-9.02)	48 (35.8)	86 (64.2)	3.45 (2.25 – 5.29)	63 (57.3)	47 (42.7)	5.81 (3.79-8.89)	51 (38.1)	83 (61.9)	3.44 (2.26 – 5.22)
	**Normal**	107 (16.4)	544 (83.6)	1.00	78 (13.9)	483 (86.1)	1.00	122 (18.7)	529 (81.3)	1.00	85 (15.2)	476 (84.8)	1.00
HDL	Abnormal	14 (73.7)	5 (26.3)	10.87 (3.85-30.68)	13 (61.9)	8 (38.1)	8.06 (3.26 – 19.91)	15 (78.9)	4 (21.1)	12.58 (4.12-38.42)	13 (61.9)	8 (38.1)	7.27 (2.95 – 17.94)
	**Normal**	148 (20.5)	575 (79.5)	1.00	113 (16.8)	561 (83.2)	1.00	166 (23.0)	557 (77.0)	1.00	123 (18.2)	551 (81.8)	1.00
Waist circumference	Abnormal	140 (46.5)	161 (53.5)	14.51 (9.20-22.90)	108 (43.0)	143 (57.0)	20.51 (12.04 – 34.95)	151 (50.2)	150 (49.8)	12.61 (8.32-19.12)	115 (45.8)	136 (54.2)	19.56 (11.84-32.34)
	**Normal**	26 (5.7)	434 (94.3)	1.00	18 (3.6)	489 (96.4)	1.00	34 (7.4)	426 (92.6)	1.00	21 (4.1)	486 (95.9)	1.00
BP	Abnormal	98 (46.4)	113 (53.6)	6.14 (4.24-8.90)	81 (36.8)	139 (63.2)	6.34 (4.21 – 9.56)	108 (51.2)	103 (48.8)	6.44 (4.48-9.24)	83 (37.7)	137 (62.3)	5.62 (3.79-8.35)
	**Normal**	68 (12.4)	482 (87.6)	1.00	45 (8.4)	490 (91.6)	1.00	77 (14.0)	473 (86.0)	1.00	52 (9.7)	483 (90.3)	1.00

AHA-NHLBI: American Heart Association and the National Heart Lung and Blood Institute; ATP III: Third Adult Treatment Panel; IDF: International Diabetes Federation; NCEP-ATP III: National Cholesterol Education Program–Third Adult Treatment Panel.

*P-value for the education trend in 2017 according to ATP III (< 0.001). P-value for trend education in 2009 according to ATP III (0.001).

*P-value for the education trend in 2017 according to NCEP-ATP III (<0.001). P-value for trend education in 2009 according to NCEP- ATP III (0.001).

There was an interaction between time and TG according to the AHA/NHLBI index (P<0.001). The odds ratio of Mets based on TG (abnormal vs. normal) was 4.67 times greater in 2017 than in 2009. Similar scenarios were found for NCEP and ATP III indexes. The odds ratio of Mets based on glucose levels (abnormal vs. normal) was 4.41 times greater in abnormal TG levels than normal TGs (P=0.01) according to the NCEP index. This odds ratio was 5.17 (P=0.01) for the ATP III index. The odds ratio of Mets was 0.28 times smaller in females compared to males in 2017 than in 2009 (P=0.007) (Table 7).

**Table 7:** Predictive factors of metabolic syndrome.

Variable		AHA/NHLBI		IDF		NCEP		ATP III	
		**P-value**	OR (95%CI) Multivariate	P-value	OR (95%CI) Multivariate	P-value	OR (95%CI) Multivariate	P-value	OR (95%CI) Multivariate
Time		0.01	1.68 (1.10 – 2.57)	-	-	0.05	1.59 (0.98 – 2.57)	0.04	1.66 (1.02 -2.69)
Sex (Female vs Male)		-	-	-	-	-	-	<0.001	3.43 (2.08-5.66)
Education	Illiterate	-	-	<0.001	4.04 (2.69 – 6.08)	-	-	-	-
	**Below high school**			<0.001	3.05 (2.11 – 4.41)				
	**Diploma**			0.002	2.10 (1.32 -3.35)				
	**Degree**			1.00					
Glucose (Abnormal vs. normal)		<0.001	13.67 (8.91-20.96)	<0.001	6.95 (5.15 -9.38)	<0.001	30.52 (16.03-58.10)	<0.001	36.97 (17.51 -78.06)
TG (Abnormal vs. normal)		<0.001	10.50 (6.68 -16.50)	<0.001	4.80 (3.47 – 6.64)	<0.001	12.08 (6.46-22.58)	<0.001	20.76 (9.81-43.95)
BP (Abnormal vs. normal)		<0.001	11.99 (8.25-17.42)	-	-	<0.001	13.62 (8.73-21.24)	<0.001	15.52 (9.78 -64.62)
Waist circumference (Abnormal vs. normal)		<0.001	40.31 (22.86 – 71.05)	-	-	<0.001	58.51 (33.62-101.85)	<0.001	36.17 (20.67 -63.29)
HDL (Abnormal vs. normal)		-	-	-	-	<0.001	26.11 (8.55-79.69)	-	-
Time by TG		<0.001	4.67 (1.99-10.98)	-	-	0.005	4.07 (1.54-10.76)	0.01	3.31 (1.22 – 9.00)
Glucose by TG		-	-	-	-	0.01	4.41 (1.37 -14.23)	0.01	5.17 (1.30 -20.52)
Time by Sex		-	-	-	-	-	-	0.007	0.28 (0.11 – 0.71)

AHA-NHLBI: American Heart Association and the National Heart Lung and Blood Institute; ATP III: Third Adult Treatment Panel; IDF: International Diabetes Federation; NCEP-ATP III: National Cholesterol Education Program—Third Adult Treatment Panel.

## Discussion

The present study demonstrated that the prevalence of Mets among the population during a period of 8 years increased by 5.2% to 7.3% based on four definitions. Accordingly, more than one-fifth of the general population in the surveyed area was currently living with Mets. Importantly, the growing trend of Mets was prominent among females than males, as well as individuals aged <40 years compared to those ≥40 years. In addition, the prevalence of Mets has risen more than 3 times in illiterate people compared to those with an academic degree. Interestingly, two components of Mets, including abdominal obesity and diabetes, increased in prevalence, whereas elevated blood pressure, hypertriglyceridemia, and low HDL, declined. Notably, a change in the prevalence trend of Mets components differed between males and females during the study period as well.

This study revealed that Mets increased annually by approximately 0.65% to 0.91% over 8 years from 2009 to 2017 in the South-East of Iran. In line with the results of this study, an increasing trend of Mets has already been reported by other studies in the United States, Korea, and Iran [9, 13, 14]. This increasing trend may be the result of an increase in life expectancy and the rapid growth of population and urbanization, as well as the adoption of a Western lifestyle [2].

A notable increase in the prevalence of Mets occurred among women (0.75% to 0.95% per year) compared to men (0.13% to 0.46% per year) during the study period, which is comparable to the findings of other studies [15]. The increase in the prevalence of Mets, especially in women, might be due to factors such as the post-menopausal period, which is usually associated with a decrease in estrogen levels [16]. It may also be associated with an increase in obesity and lack of physical exercise in Iranian women [17].

Data suggested a reverse association between the level of education and the prevalence trend of Mets such that individuals with a low level of education were more likely to be affected by Mets. These findings are in line with the Korean study in which Mets risk decreased in women with higher education levels [18]. An explanation for this might be the fact that academic and educated people could be more aware of inactivity, unhealthy food patterns, and risky behaviors. Accordingly, they are more likely to take care of themselves by doing systematic exercise, consume healthy food, and avoid risky behaviors such as smoking and drinking, as well as orderly health check-ups.

As expected, Mets was more prevalent in individuals aged ≥40 years than those aged <40 years in both study periods regardless of the definition, which is comparable with reports that come from other studies across the world [19, 20]. Nevertheless, the increasing trend of Mets from 2009 to 2017 was considerably higher in younger (< 40 years) than older (≥40 years) people. A higher increase in the young may be explained by the increasing trend of obesity as well as the popularity of the Western lifestyle in these age groups.

In this study, the frequency of blood glucose and central obesity had an increasing trend in both sexes and a greater increase in women. These two components account for much of the escalation in the frequency of Mets based on reports coming from other studies as well [3, 21]. The increase in blood glucose and central obesity may be attributed to the changes in lifestyle, the popularity of the Western diet, and reduction in physical activity in the studied population [22], which is similar to the findings of other studies [1, 23]. For example, most countries across the world, including Iran, are now facing the obesity epidemic because of the aforementioned reasons [24]. On the other hand, there is some evidence that obesity is closely associated with type 2 diabetes and Mets.

While the prevalence of elevated blood pressure has declined slightly over time, there have been divergent trends by gender. Indeed, the prevalence of elevated blood pressure increased among women by 3.4% during the study period. In contrast, there was a downward trend of 5.4% in elevated blood pressure among men over time. Our results demonstrate a notable decrease in low HDL-cholesterol levels over time, which was more pronounced in females, as stated by other authors [23]. There is some evidence that increased consumption of drugs and the use of hookah among target populations might be one of the reasons for lower HDL levels in their blood [25, 26].

Also, data suggested a descending trend in the prevalence of TG among men while it remained relatively stable in women. This disparity might be related to lifestyle risk factors including a reduction in physical activity, unhealthy diet, obesity, dyslipidemia, and medical interventions in this period (e.g., taking blood pressure, lipid-lowering medications, change in the level of blood insulin, and other physiological and environmental factors [23, 27].

In the present study, blood sugar, waist circumference, blood pressure, and low HDL, as well as sex and education, were predicting factors of Mets that were similar to other studies [28-30]. Weight gain is associated with insulin resistance and Mets. On the other hand, weight loss is effective in improving glucose tolerance and reducing type 2 diabetes [31]. In addition, studies have shown an association between abdominal obesity and high blood pressure as well as hyperlipidemia [8, 32]. Moreover, elevated triglyceride levels, especially when combined with low HDL levels, can replace insulin resistance and ultimately be effective in the development of Mets [33]. This relationship between the above-mentioned variables might be the explanation for their predicting role in Mets.

However, the present study has some limitations. The entire population that was studied in 2009 was not available in 2017. Additionally, population movements are high in this area. Lack of their annual follow-up is another disadvantage of the study. Nevertheless, missing cases were approximately distributed in all clusters. Therefore, the sample can be a good representative of the inhabitants of Zahedan, a less developed and deprived region of the country.

Moreover, the study was conducted in the South-Eastern part of Iran, a city at the border of Pakistan and Afghanistan, with specific ethnicities. Therefore, these findings could be of interest to health policymakers of national and neighboring countries.

## Conclusion

This study revealed an increasing trend of Mets in a less developed region of Iran. Moreover, the growing trend of some components of Mets over an eight-year interval highlights the need for preventive interventions such as a healthy diet and lifestyle as well as physical activity to reduce the level of these components, e.g., waist circumference and blood glucose.

## Acknowledgment

This manuscript is a part of the Ph.D. thesis of Khadijeh Kalan Farmanfarma, which has been funded by a scholarship from the Zahedan University of Medical Sciences (Grant no: 8140).

## Conflict of Interest

The authors declare that there is no conflict of interest.
